# The association of copy number variation and percent mammographic density

**DOI:** 10.1186/s13104-015-1212-y

**Published:** 2015-07-08

**Authors:** Elizabeth J Atkinson, Jeanette E Eckel-Passow, Alice Wang, Alexandra J Greenberg, Christopher G Scott, V Shane Pankratz, Kristen N Purrington, Thomas A Sellers, David N Rider, John A Heit, Mariza de Andrade, Julie M Cunningham, Fergus J Couch, Celine M Vachon

**Affiliations:** Department of Health Sciences Research, Mayo Clinic, 200 First Street SW, Rochester, MN 55905 USA; Wayne State University School of Medicine and Karmanos Cancer Institute, Detroit, MI USA; Department of Cancer Epidemiology, Moffitt Cancer Center, Tampa, FL USA; Division of Cardiovascular Disease, Department of Medicine, Mayo Clinic, Rochester, MN USA; Department of Molecular Pharmacology and Experimental Therapeutics, Mayo Clinic, Rochester, MN USA

**Keywords:** Breast density, Mammographic density, Genetics, Copy number variation

## Abstract

**Background:**

Percent mammographic density (PD) estimates the proportion of stromal, fat, and epithelial breast tissues on the mammogram image. Adjusted for age and body mass index (BMI), PD is one of the strongest risk factors for breast cancer [1]. Inherited factors are hypothesized to explain between 30 and 60% of the variance in this trait [2–5]. However, previously identified common genetic variants account for less than 6% of the variance in PD, leaving much of the genetic contribution to this trait unexplained. We performed the first study to examine whether germline copy number variation (CNV) are associated with PD. Two genome-wide association studies (GWAS) of percent density conducted on the Illumina 660W-Quad were used to identify and replicate the association between candidate CNVs and PD: the Minnesota Breast Cancer Family Study (MBCFS) and controls from the Mayo Venous Thromboembolism (Mayo VTE) Case–Control Study, with 585 and 328 women, respectively. Linear models were utilized to examine the association of each probe with PD, adjusted for age, menopausal status and BMI. Segmentation was subsequently performed on the probe-level test statistics to identify candidate CNV regions that were associated with PD.

**Results:**

Sixty-one probes from five chromosomal regions [3q26.1 (2 regions), 8q24.22, 11p15.3, and 17q22] were significantly associated with PD in MBCFS (p-values <0.0001). A CNV at 3q26.1 showed the greatest evidence for association with PD; a region without any known SNPs. Conversely, the CNV at 17q22 was largely due to the association between SNPs and PD in the region. SNPs in the 8q24.22 region have been shown to be associated with risk of many cancers; however, SNPs in this region were not responsible for the observed CNV association. While we were unable to replicate the associations with PD, two of the five CNVs (3q26.1 and 11p15.3) were also observed in the Mayo VTE controls.

**Conclusions:**

CNVs may help to explain some of the variability in PD that is currently unexplained by SNPs. While we were able to replicate the existence of two CNVs across the two GWAS studies, we were unable to replicate the associations with PD. Even so, the proximity of the identified CNV regions to loci known to be associated with breast cancer risk suggests further investigation and potentially shared genetic mechanisms underlying the PD and breast cancer association.

**Electronic supplementary material:**

The online version of this article (doi:10.1186/s13104-015-1212-y) contains supplementary material, which is available to authorized users.

## Background

Percent mammographic density (PD) is an estimate of the proportion of stromal and epithelial breast tissues on the mammogram image. Adjusted for age and body mass index (BMI), PD is one of the strongest risk factors for breast cancer, and women in the highest quartile of density have a 3- to 5-fold increased risk compared to women in the lowest quartile [1]. Twin and family studies have shown that PD is highly heritable and that inherited factors are estimated to explain between 30 and 60% of the variance in this trait [2–5]. To date, several genetic loci or single nucleotide polymorphisms (SNPs) have been identified to be associated with percent density using genome-wide association studies (GWAS), including a novel locus on chromosome 12q24 and established breast cancer loci, *ZNF365, ESR1, LSP1* and *RAD51L1*, suggesting shared heritability between PD and breast cancer [6–8]. However, together these SNPs are estimated to account for less than 6% of the variance in PD, leaving much of the genetic contribution to this trait unexplained.

Like SNPs, the deletion or amplification of segments of DNA, known as copy number variation (CNV), are common in the germline and have been implicated in the risk of diseases including neuroblastoma, cataracts, and cancer [9–13]. In fact, CNVs are estimated to account for 13% of the human genome [9, 14, 15]. While the mechanisms underlying the development of CNVs remain generally unknown, it has been shown that CNVs are frequently located near telomeres, centromeres, and proximal duplicated regions [9, 16, 17]. Furthermore, rare germline genomic duplications and deletions have been shown to disrupt high-penetrance tumor suppressor genes, such as the *BRCA1* and *BRCA2* genes, in breast cancer patients, and have been demonstrated to aggregate within families [18–20]. Several recent publications have linked germline copy number variation (CNV) in other regions of the genome, including both inter- and intra-genic regions, with risk or recurrence of breast cancer [21–24].

As PD has been shown to be highly heritable, we hypothesized that some of the variance not explained by associated SNPs could be due to germline CNVs. CNVs have been shown to have adequate coverage on current SNP arrays, at least for large and intermediate size CNVs (CNVs >5 kb) [17], and the size of identified deletions and amplifications in most of the prior studies with cancer ranged from intermediate (4 kb) to large (2 Mb). Therefore, using data from two independent GWAS studies, we performed the first study to examine whether CNVs are associated with PD.

## Methods

### Subjects

Two independent studies contributed copy number and PD phenotype information. The protocol was approved by the Mayo Clinic Institutional Review Board. The first stage utilized 595 women of white European ancestry with GWAS and PD data from the Minnesota Breast Cancer Family Study (MBCFS) [6, 25, 26]. Briefly, females from 89 multigenerational families ascertained through a breast cancer proband diagnosed between 1944 and 1952 and who provided the location and consent to retrieve their mammograms were recruited to a family study of breast density. Among the 737 age-eligible women (over age 40) we retrieved the mammograms of 658 (89%). Of these, 595 women had DNA available for GWAS analyses [6].

The replication stage consisted of 336 women who were female controls within the Mayo Venous Thromboembolism Case–Control Study (Mayo VTE) [6, 27]. Clinic-based controls were prospectively selected from persons undergoing outpatient general medical examinations from 2004 to 2009 who had no previous diagnosis of VTE or superficial vein thrombosis, active cancer, antiphospholipid antibody syndrome, rheumatologic or other autoimmune disorder, or prior bone marrow or liver transplant.

Both populations were genotyped on the Illumina 660W-Quad genotyping platform, which provided information on 657,172 autosomal probes for the evaluation of CNVs.

For both studies, the mammogram closest to enrollment date was obtained and digitized on either a Lumiscan 75 scanner (MBCFS) or Array 2905HD Laser Film Digitizer (Mayo VTE). PD was estimated by the same programmer (FFW) using a computer-assisted thresholding program Cumulus [28]. For MBCFS, percent density from the mediolateral oblique and craniocaudal views were averaged and used as the primary phenotype and for Mayo VTE, only the left craniocaudal view was used. We have previously shown concordance of density from both breast sides and views [4]. Although both studies had high intrareader reliability (>0.9 for both), we acknowledge the lower PD in the Mayo VTE population that is partly due to the increased age and BMI of the women relative to MBCFS, but also due to drift in the PD measure with time. There were 5 years between evaluations of PD for these two studies. However, these two studies both identified significant associations with a SNP at chromosome 12 [6] and show similar associations with clinical characteristics (data not shown).

### Statistical analysis

Log R ratio (LRR) data were extracted from the two GWAS using Genome Studio. The LRR data for each probe were median normalized per plate so that the distributions of LRR values were similar across all plates [29, 30]. PennCNV was used to extract quality-control metrics and samples were removed from further analysis if the standard deviation of the LRR >0.35, the B-Allele Frequency (BAF) drift was >0.0015, the wave factor was >0.05, or the number of CNV intervals was >500 [31].

The primary goal of the analysis was to identify copy number regions associated with PD. To do so, we first utilized the probe level data and performed probe-specific tests using linear mixed effects models for MBCFS (to account for the family design) and linear models for Mayo VTE (Flow diagram, Additional file 1) [32]. The square-root of PD was the dependent variable and probe-specific LRR values, age, inverse of body mass index (BMI) and menopausal status were included as independent variables. Second, we identified candidate copy number regions of interest by applying circular binary segmentation (CBS) to the absolute value of the probe-specific test statistics [33]. The absolute values of the probe-specific test statistics were averaged within each segment. Segments defined by three or more probes with a mean test statistic greater than one were considered for further analysis. Third, we conservatively expanded the segments by including six times the initial number of probes in the CBS identified segment both prior to the start of the segment and after the end of the segment. For example, if a segment contained ten probes, then the expanded region would add 60 probes to the start and an additional 60 probes to the end of the segment for a total size of 130 probes. Empirically this appeared to be a sufficient expanded region so as to not impact the identification of the segment of interest. Fourth, we applied permutation tests to each expanded region using 10,000 iterations. For each iteration the phenotype was permuted, the probe-specific association models (mixed effect model for MBCFS and linear model for Mayo VTE) were run, and the CBS algorithm was applied. The probe-specific test statistics for all probes within the CBS identified region were averaged and the region was assigned the mean value. For identified regions that included a significant SNP, the above modeling process was repeated including the most significant SNP and the LRR value.

P values were computed from the permutation tests and were based on how many times the observed, non-permuted test statistic, exceeded all of the permuted runs (p value = N/10,001). If there were no permuted observations greater than the observed value, then the probe was assigned the value 1/10,001. Any given probe was considered to be significantly associated with breast density if the permuted P < 1/10,000. For the replication analysis, a significance level of 0.05 was used.

Recent projections suggest that CNVs may account for 13% of the human genome and their occurrences have been cataloged in public databases such as the Toronto Database of Genomic Variants and the Genome Structural Variation Consortium CNV discovery project [9, 14, 15]. As a secondary analysis, we evaluated whether any of our primary identified regions were in this database. We then used the validation-calling algorithm within PennCNV, which is designed to call CNVs in known common CNV regions by using all the probes within a defined region and identifying the most likely copy number (0, 1, 2, 3 and 4) [31]. Association analyses (mixed effect model for MBCFS and linear model for Mayo VTE) were run to test the association of common CNVs with PD.

Each SNP (coded as 0, 1, or 2) was also evaluated using a linear model (linear mixed effects model for MBCFS) where the square-root of PD was the dependent variable and SNP, age, inverse of body mass index (BMI) and menopausal status were included as independent variables.

## Results

The evaluation of the log R ratio (LRR) standard deviation, B-allele frequency (BAF) drift and wave factor for the 595 members of the Minnesota Breast Cancer Family Study (MBCFS) resulted in exclusion of ten subjects (Additional file 2). Thus, 585 patients were analyzed to identify associations between CNV and PD, adjusted for age, BMI and menopausal status. PD was lower in the Mayo VTE population, partly due to the slightly older age and higher BMI, but also due to drift in the PD measure can occur with time (Table 1).

**Table 1:** Characteristics of subjects used in the discovery and replication phases

Study	MBCFS	Mayo VTE
Design	Family study	Case–control study
Cases/ controls	0/585	0/328
Age, mean (SD) (years)	57.2 (11.6)	61.0 (12.7)
BMI, mean (SD) (kg/m^2^)	27.1 (5.7)	28.4 (6.1)
Pre-menopausal (%)	30.4	25.6
Percent density, mean (SD)	26.6 (15.91)	14.6 (13.62)
Mammogram view	Average of CC and MLO	CC
PD measurement software	Cumulus	Cumulus
Digitizer Software	Lumiscan	Array 2905

*MBCFS* Mayo Breast Cancer Family Study and *Mayo VTE* venous thromboembolism Case–Control Study. *CC* Craniocaudal and *MLO* mediolateral oblique.

**Figure 1:**
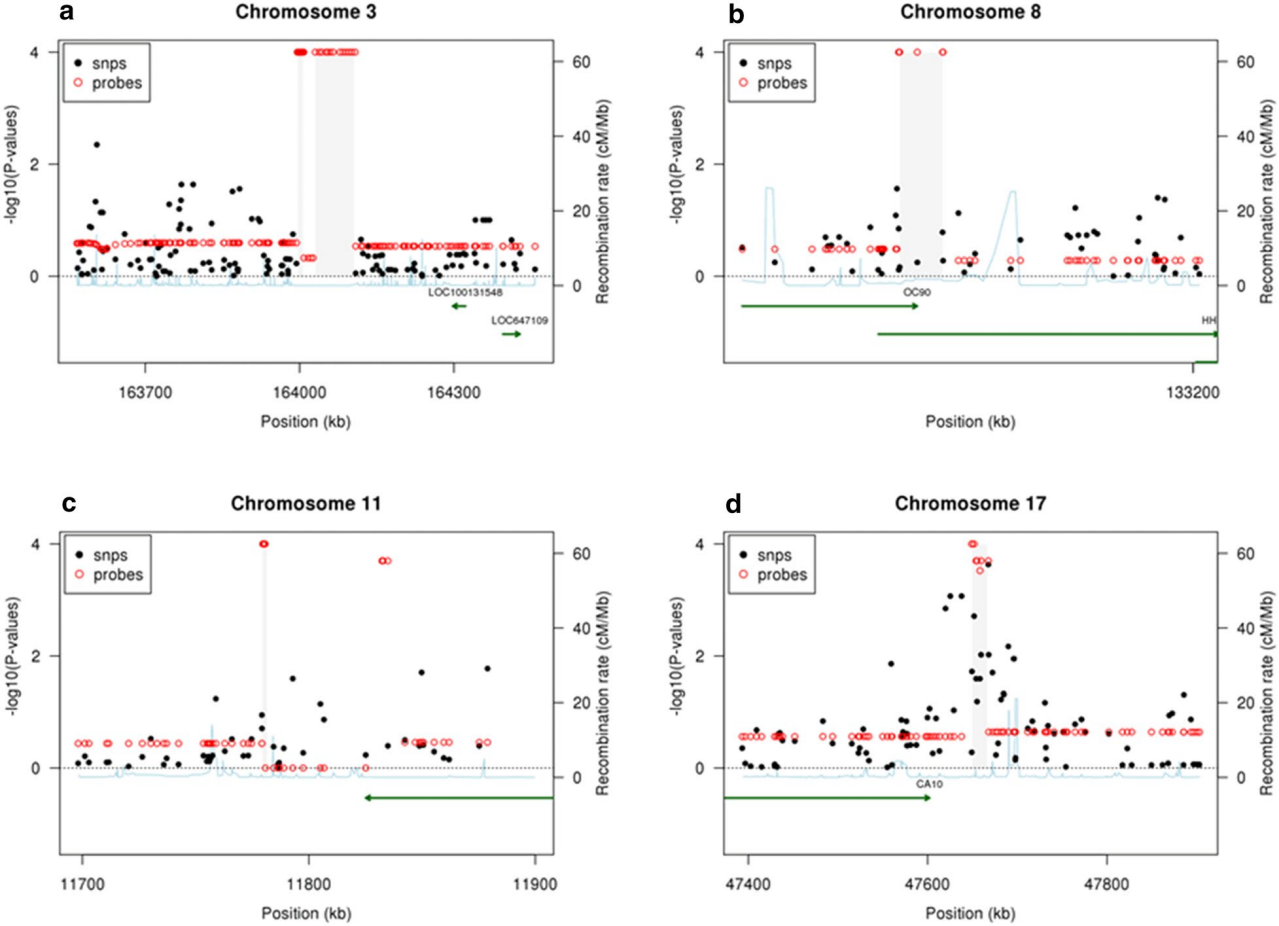
Candidate CNVs and SNP associations with PD in the Mayo Breast Cancer Family Study (MBCFS) for **a** 3q26.1 [2 regions], **b** 8q24.22, **c** 11p15.3 and **d** 17q22. For identifying candidate CNVs that are associated with PD, we performed probe-specific tests and subsequently performed segmentation on the test-statistics. P values were computed from permutation tests and were based on how many times the observed test-statistic, exceeded the permutation test statistics (using 10,000 permutations). *Red circles* denote CNV probes, *black dots* denote SNPs, the *blue line* denotes recombination rate, *green lines* denote genes, and the *grey shaded areas* denote the CNV region that was observed in MBCFS.

**Figure 2:**
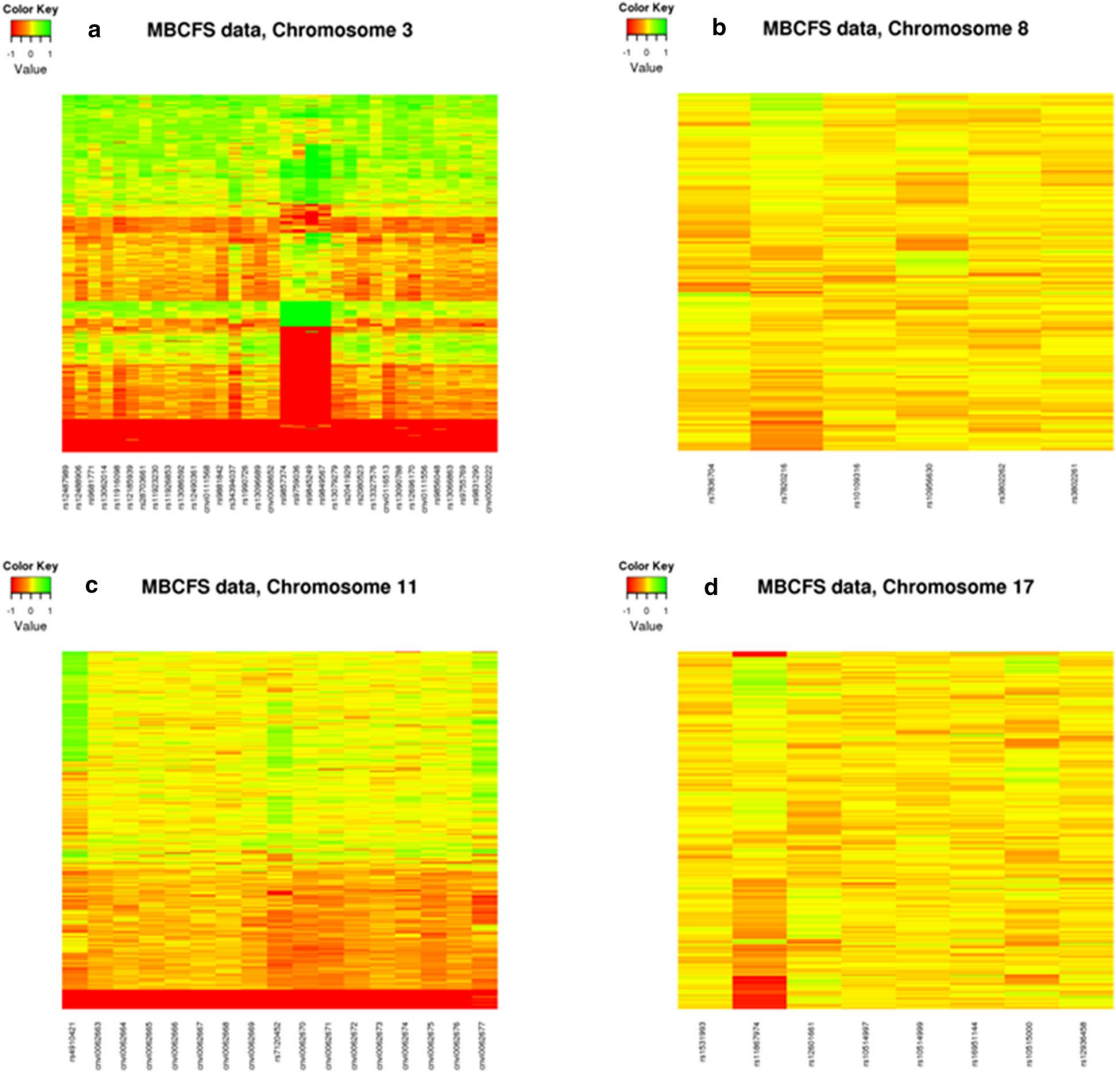
LRR values from the Mayo Breast Cancer Family Study (MBCFS) for **a** 3q26.1 [2 regions], **b** 8q24.22, **c** 11p15.3, and **d** 17q22. Each *row* represents a sample and each *column* represents a probe.

Analysis of the 657,172 autosomal chromosome probes from the Illumina 660W Quad identified five regions on four chromosomes [3q26.1 (contained two regions), 8q24.22, 11p15.3, and 17q22] to be significantly associated with PD after adjusting for age, menopausal status, and BMI in MBCFS (Table 2). Candidate regions identified in the initial data reduction analysis step are shown in Additional file 3. Figure 1 shows the probe-level p-values, SNP p-values, recombination rates, and neighboring genes for each of the four chromosomes. Figure 2a–d shows the LRR values for each of the four chromosomes. The two 3q26.1 regions consisted of a total of 30 probes that were significantly associated with PD in MBCFS and were clustered in a region without SNPs on the Illumina 660W platform (Figure 1a). Our algorithm detected two deleted CNV regions at 3q26.1 that were associated with PD in MBCFS. These two regions were separated by a segment defined by four probes that contains both insertions and deletions (Figure 2a). The 8q24.22 region consisted of 6 probes that were significantly associated with PD in MBCFS (Figure 1b). This region contains two genes: *HHLA1* and *OC90*. Upon closer examination of the LRR values, it appears that a single probe (SNP) was driving the association results (Figure 2b). However, while there are SNPs in this region, none were significantly associated with PD (Figure 1b). The 11p15.3 region consisted of 17 probes that were significantly associated with PD in MBCFS (Figure 1c). These 17 probes defined a deleted region on 11p15.3 (Figure 2c). And, while there were SNPs in this region, none were significantly associated with PD (Figure 1c). The 17q22 region consisted of three probes that were significantly associated with PD in MBCFS (Figure 1d). In contrast to the other regions, the 17q22 region had SNPs that were significantly associated with PD; the most significant SNP was rs12936458 (*p* value = 0.00023). After adjusting for rs12936458, the CNV probes were no longer associated with PD.

**Table 2:** CNV Regions identified in discovery phase (MBCFS) and evaluated in replication cohort (Mayo VTE)

Chromosome	Start position	End position	Number significant probes^a^	
			MBCFS (discovery)	Mayo VTE (replication)
3	163,995,377	164,008,284	17	1
3	164,030,569	164,108,060	13	0
8	133,134,063	133,144,009	6	0
11	11,779,614	11,780,713	17	0
17	47,649,105	47,667,700	3	3

*MBCFS Mayo* Breast Cancer Family Study and *Mayo VTE* Mayo Venous Thromboembolism Case–Control Study.

^a^Significant associations defined as p < 0.0001 for discovery phase and p < 0.05 for replication.

**Figure 3:**
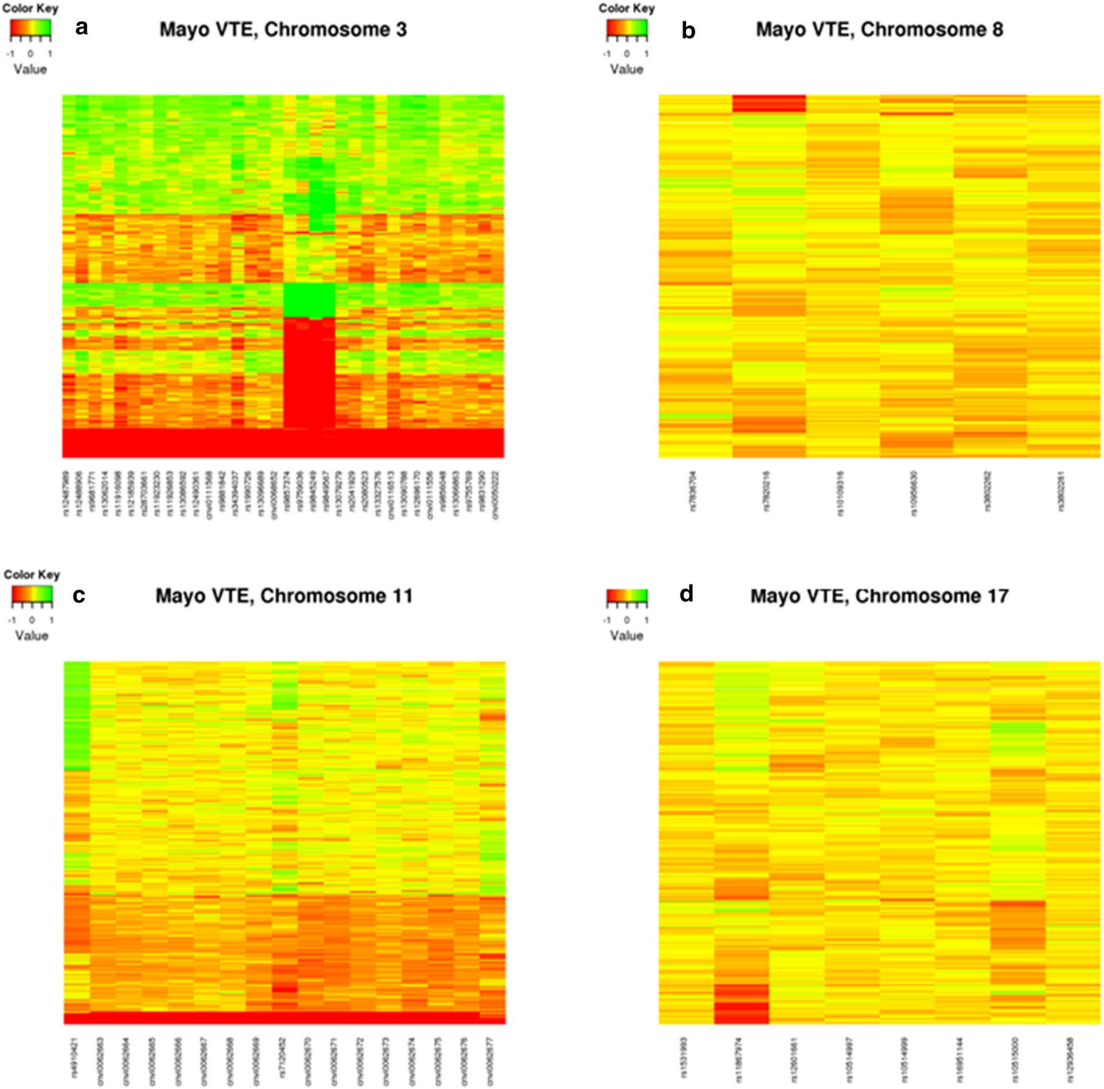
LRR values from the Mayo VTE control samples for **a** 3q26.1 (2 regions), **b** 8q24.22, **c** 11p15.3, and **d** 17q22. Each *row* represents a sample and each *column* represents a probe.

The Mayo VTE controls were used for replication. Similar quality-control exclusions were made for the Mayo VTE controls, resulting in 328 individuals being analyzed (Table 1; Additional file 2). As demonstrated by Figure 3, the Mayo VTE controls showed similar CNVs for the two regions at 3q26.1 (Figure 3a) and the region at 11p15.3 (Figure 3c) as was observed in MBCFS. Alternative versions of these figures for both studies are shown in Additional files 4, 5, 6 and 7. While the CNVs were observed across the two datasets, we did not observe an association with PD in MBCFS. Specifically, only one probe at 3q26.1 showed a significant association with PD in the Mayo VTE controls (p < 0.0001 Table 2; Additional file 8); however, the coefficient was of the opposite direction (Additional file 9).

As mentioned previously, the two 3q26.1 regions are in an area on the Illumina 660W Quad array that only contained CNV probes (there were no SNPs in this region; Figure 1a), suggesting that this is a known CNV region. This was confirmed by the absolute, inter-valued copy number estimates for 450 HapMap samples obtained from the Genome Structural Variation Consortium CNV discovery project, where chromosome 3 (163,994,833–164,109,307) is listed [9]. This particular region showed 54 (30%) CEU HapMap samples with deletions (18 with zero copies, 36 with one copy) and 60 (34%) samples with insertions. Thus, using PennCNV thirty-four probes in this region were used to force a CNV call in both the MBCFS and Mayo VTE samples. Within MBCFS there were 181 (31%) samples with deletions (54 with zero copies, 127 with one copy) and 13 (2%) samples with insertions. The number of deletions was similar to those detected in the HapMap samples; however, the insertions were significantly less frequent in the MBCFS cohort. Comparison of samples with a deletion versus those without a deletion in MBCFS was significantly associated with PD (p = 0.005). The Mayo VTE control samples were used for replication: 10 (3%) subjects had deletions (three with zero copies, seven with one copy) and no samples had an insertion. Thus, the Mayo VTE controls did have similar number of deletions and insertions and we did not observe a significant association with PD.

## Discussion

Prior reports have demonstrated that PD is a heritable trait; however, to date, only a small percentage of PD variation is explained by SNPs. To determine whether CNVs account for some of the remaining variability we conducted the first analysis of CNV and PD using two previously-published GWAS datasets. In the discovery set (MBCFS) we identified five candidate regions that were significantly associated with PD: 3q26.1 [2 regions], 8q24.22, 11p15.3 and 17q22.

A CNV at 3q26.1 (163,698,399 to 163,718,292 in GRCh36/hg18; near the one we identified in the present study) has been previously reported to be associated with breast cancer risk in a Japanese population, though the start and end positions are slightly different [23]. We observed two CNVs at this region in both MBCFS and Mayo VTE control samples. However, only a single probe in the 3q26.1 region was observed to be associated in the Mayo VTE replication samples and the effect was in the opposite direction. This discrepancy could be due to the fact that the two datasets had different study designs (MBCFS is a family study and the Mayo VTE controls were obtained from a case–control study) and/or because the PD distributions are different across the two studies. Because the CNV region replicates and because an association with breast cancer risk has been previously reported [23], we suggest that further investigation needs to be undertaken to further try to replicate the result observed in MBCFS. Notably, neither the CNV region identified in the Japanese study or our study mapped to a gene [34, 35]. Lastly, it is important to note that the 3q26.1 regions would not be detected via GWAS analysis using the Illumina 660 W-Quad because there are no known SNPs in this region.

The 8q24 region has been shown to be associated with risk of many cancers, including breast, [36–42]. While our algorithm detected a CNV at 8q24.22 that was associated with PD in the MBCFS samples, we were not able to replicate the results in Mayo VTE. Furthermore, upon further evaluation of the data, it appears that the results are largely being driven by a single probe. Even so, because the 8q24 region has been previously reported, we suggest that further investigation should be undertaken to replicate these results.

Our algorithm identified a CNV at 11p15.3 in MBCFS, which was validated in the Mayo VTE samples. However, we only observed a significant association with PD in the MBCFS samples. To our knowledge, this region has not been identified previously.

A CNV region at 17q22 was identified to be significantly associated with PD in MBCFS. However, we determined that the association between this region and PD was driven by known SNPs in the region. Particularly, after adjusting for the most significant SNP in this region (rs12936458) the CNV was no longer associated with PD. Even so, the 17q22-23 region has previously been implicated in breast cancer risk, and also has been shown to have copy number abnormalities and amplification in breast cancer cell lines and tumors [43–45]. Additionally, CNVs significantly associated with breast cancer risk have been previously reported in the adjacent 17q21 region, at *BRCA1* [18, 20]. Therefore, additional examination of 17q21-23 may elucidate the role of CNVs in breast cancer pathogenesis.

Our analysis approach involved performing statistical association tests on the probe-level data and subsequently performing segmentation on the probe-level test statistics to identify candidate regions that are associated with PD [46]. Our approach is different from the majority of CNV analyses where candidate CNVs are first identified for each sample, the candidate CNVS are subsequently grouped together to create consensus regions across all samples, and lastly, the consensus regions are tested for associations with the trait of interest [47–49]. There are at least two problematic aspects of this approach. First, there are numerous CNV detection algorithms available and unfortunately, the consensus of these algorithms is very low [40]. Second, it is not a trivial task to define consensus regions across individuals. Thus, the strength of the approach used herein is that it avoids the variability associated with both identifying candidate CNV regions and defining common CNV regions across individuals. Motivation for our approach is shown in simulations presented by Breheny, who found that probe-level testing can offer a significant increase (>12-fold) in power over traditional CNV-level testing [46]. We acknowledge that probe-level testing can be computationally intensive; however, we minimized this issue by performing segmentation only in those regions where there was some indication of a statistical association. A potential weakness with our approach, as shown by Breheny, is that it may not perform as well when CNV are large and rare. Additionally, our approach is not as sensitive when there are duplications and insertions at the same location. These limitations notwithstanding, our probe-level testing approach identified candidate CNVs that were evident in both datasets, as shown in Figure 2.

## Conclusion

In summary, we identified five candidate CNV regions [3q26.1 (2 regions), 8q24.22, 11p15.3 and 17q22] that showed evidence of an association with PD. While three of CNV regions were observed in a second dataset [3q26.1 (2 regions), 11p15.3], we were unable to replicate the associations with PD. However, these CNVs have been previously implicated in breast cancer risk as well as other malignancies. Thus, there is a possibility that they did not replicate in the Mayo VTE samples because of the different experimental design, power and/or the different PD measurements. As such, we recommend additional investigations to further examine these CNVs with PD and breast cancer in other populations to better understand genetic mechanisms by which PD may influence breast cancer risk.

## Additional files

Additional file 1: CNV analysis procedure. This flowchart represents the steps that were used to identify candidate CNV regions that are associated with PD. Additional file 2: Study-specific genotyping details for discovery (MBCFS) and replication (Mayo VTE). Additional file 3: 48 candidate regions were identified in the data reduction step of the analysis (described in Supplementary Figure 1). Subsequently, five regions were identified as having a statistically-significant association with percent density in MBCFS. Additional file 4: LRR values from the Mayo Breast Cancer Family Study (MBCFS) and the Mayo VTE control samples for 3q26.1 [2 regions]. Each line represents a sample and the colors represent different CNV patterns. Additional file 5: LRR values from the Mayo Breast Cancer Family Study (MBCFS) and the Mayo VTE control samples for 8q24.22. Each line represents a sample and the colors represent different CNV patterns. Additional file 6: LRR values from the Mayo Breast Cancer Family Study (MBCFS) and the Mayo VTE control samples for 11p15.3. Each line represents a sample and the colors represent different CNV patterns. Additional file 7: LRR values from the Mayo Breast Cancer Family Study (MBCFS) and the Mayo VTE control samples for 17q22. Each line represents a sample and the colors represent different CNV patterns. Additional file 8: Candidate CNVs and SNP associations with PD for the two regions that had significant associations in the Mayo VTE samples: (A) 3q26.1 [2 regions] and (B) 17q22.  For validating the candidate CNVs that were found to be associated with PD in the MBCFS study, we performed probe-specific tests and subsequently performed segmentation on the test-statistics in the Mayo VTE samples. P-values were computed from permutation tests and were based on how many times the observed test-statistic, exceeded the permutation test statistics (using 10,000 permutations). Red circles denote CNV probes, black dots denote SNPs, the blue line denotes recombination rate, green lines denote genes, and the grey shaded areas denote the CNV region. Additional file 9: Coefficients and permutation based p-values for chromosome 3 and 17 using the discovery (MBCFS) and replication (Mayo VTE) cohorts.

## Abbreviations

BAF: B-allele frequency
BMI: body mass index
CBS: circular binary segmentation
CNV: copy number variation
GWAS: genome-wide association studies
LRR: log R ratio
MBCFS: Minnesota breast cancer family study
PD: percent mammographic density
SNP: single nucleotide polymorphism
Mayo VTE: venous thromboembolism Case–Control Study
