# Supplementary figures and images for: The association of copy number variation and percent mammographic density
Source: BMC Res Notes. 2015 Jul 8;8:297. doi: 10.1186/s13104-015-1212-y (PMC4494822; doi:10.1186/s13104-015-1212-y)

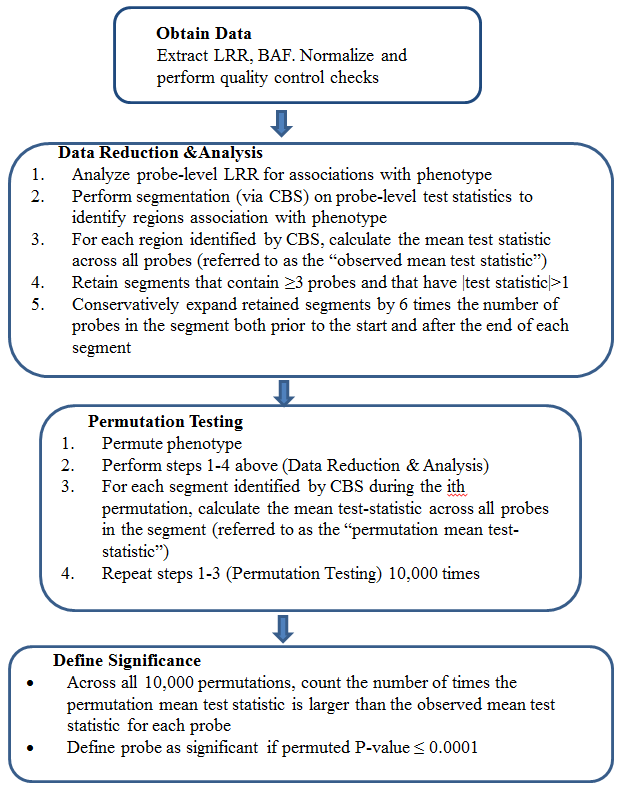

Supplement: Additional file 1: — CNV analysis procedure. This flowchart represents the steps that were used to identify candidate CNV regions that are associated with PD. [file 13104_2015_1212_MOESM1_ESM.tif]

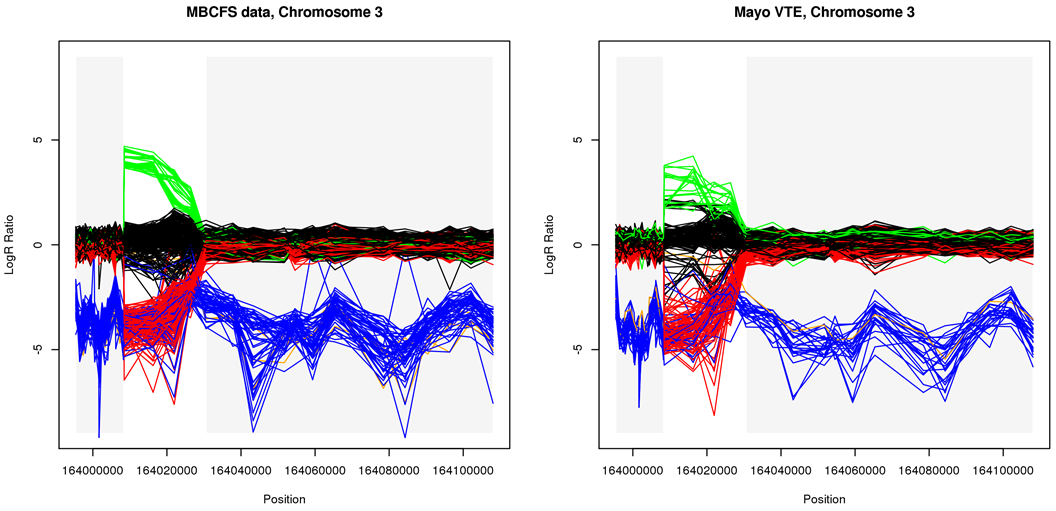

Supplement: Additional file 4: — LRR values from the Mayo Breast Cancer Family Study (MBCFS) and the Mayo VTE control samples for 3q26.1 [2 regions]. Each line represents a sample and the colors represent different CNV patterns. [file 13104_2015_1212_MOESM4_ESM.tif]

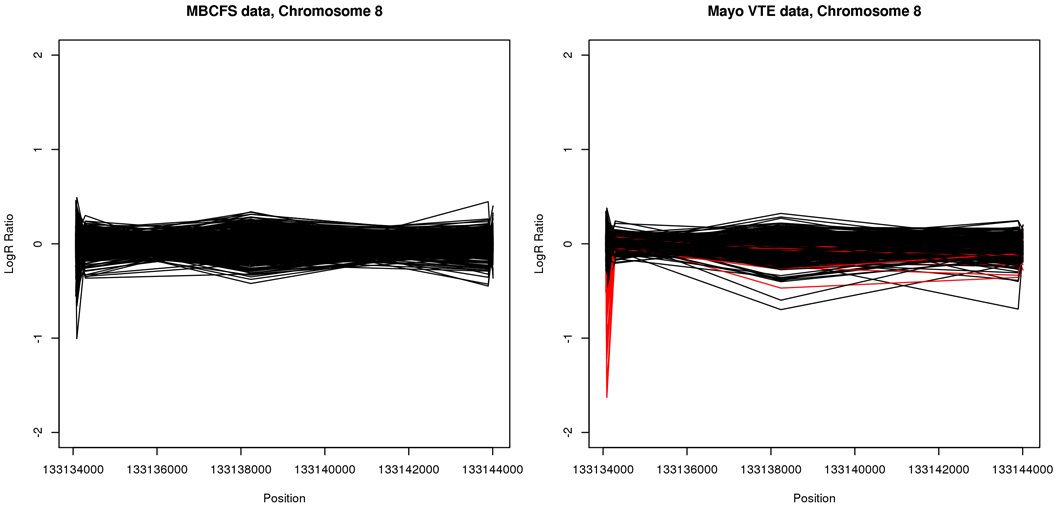

Supplement: Additional file 5: — LRR values from the Mayo Breast Cancer Family Study (MBCFS) and the Mayo VTE control samples for 8q24.22. Each line represents a sample and the colors represent different CNV patterns. [file 13104_2015_1212_MOESM5_ESM.tif]

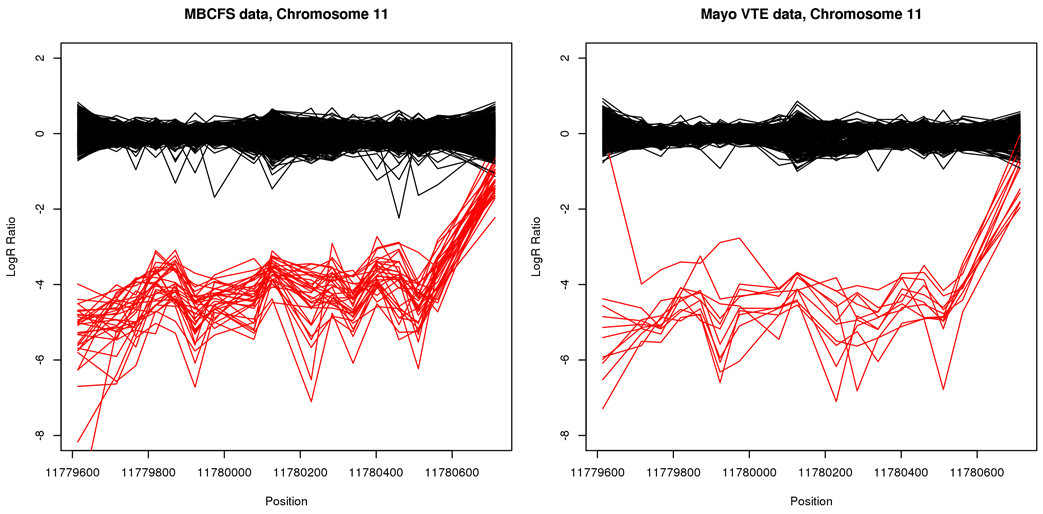

Supplement: Additional file 6: — LRR values from the Mayo Breast Cancer Family Study (MBCFS) and the Mayo VTE control samples for 11p15.3. Each line represents a sample and the colors represent different CNV patterns. [file 13104_2015_1212_MOESM6_ESM.tif]

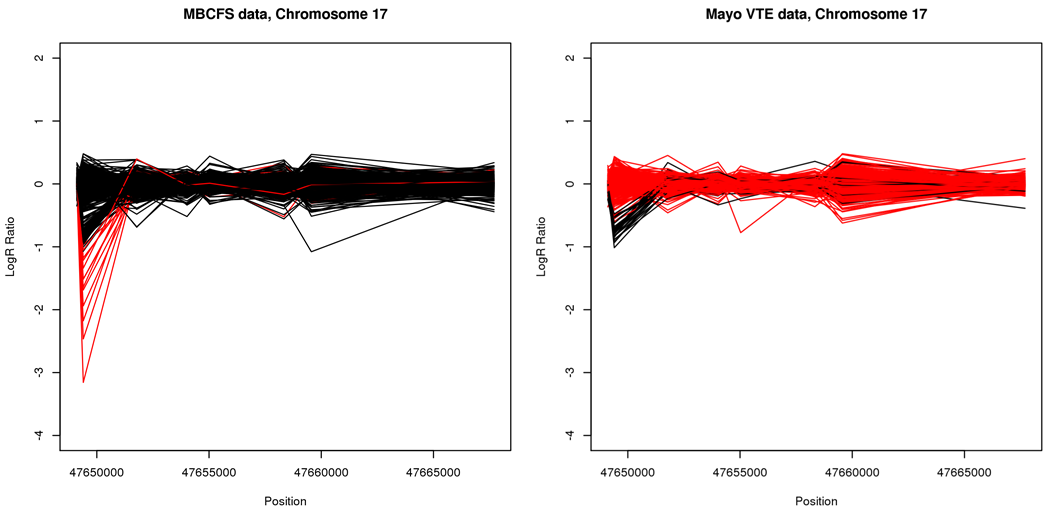

Supplement: Additional file 7: — LRR values from the Mayo Breast Cancer Family Study (MBCFS) and the Mayo VTE control samples for 17q22. Each line represents a sample and the colors represent different CNV patterns. [file 13104_2015_1212_MOESM7_ESM.tif]

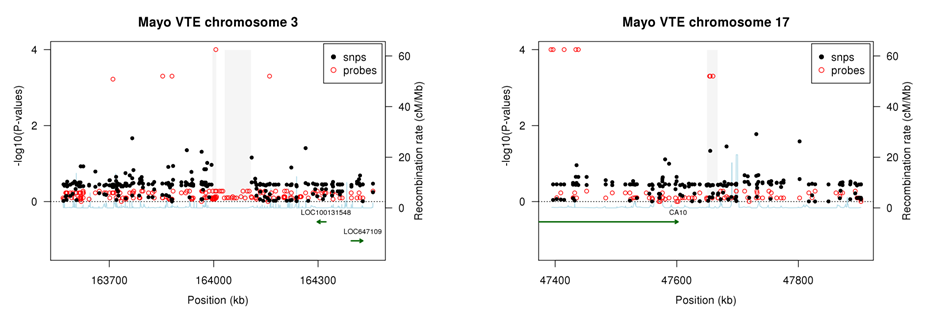

Supplement: Additional file 8: — Candidate CNVs and SNP associations with PD for the two regions that had significant associations in the Mayo VTE samples: (A) 3q26.1 [2 regions] and (B) 17q22. For validating the candidate CNVs that were found to be associated with PD in the MBCFS study, we performed probe-specific tests and subsequently performed segmentation on the test-statistics in the Mayo VTE samples. P-values were computed from permutation tests and were based on how many times the observed test-statistic, exceeded the permutation test statistics (using 10,000 permutations). Red circles denote CNV probes, black dots denote SNPs, the blue line denotes recombination rate, green lines denote genes, and the grey shaded areas denote the CNV region. [file 13104_2015_1212_MOESM8_ESM.tif]
