# Supplementary material for: The association of copy number variation and percent mammographic density
Source: BMC Res Notes. 2015 Jul 8;8:297. doi: 10.1186/s13104-015-1212-y (PMC4494822; doi:10.1186/s13104-015-1212-y)
Supplement: Additional file 2: — Study-specific genotyping details for discovery (MBCFS) and replication (Mayo VTE). [file 13104_2015_1212_MOESM2_ESM.docx]

**Supplementary Table 1.** Study-specific genotyping details for discovery (MBCFS^a^) and replication (Mayo VTE^a^)

|  |  | **MBCFS**^a^ | **Mayo VTE**^a^ |
| --- | --- | --- | --- |
|  |  | n=595 | n=336 |
| **Genotyping** | Platform | Illumina 660W Quad | Illumina 660W Quad |
|  | Facility | Mayo Clinic | CIDR |
| **Quality control** | **Sample exclusions:** |  |  |
|  | Number CNV > 500 | 3 | 4 |
|  | B-allele frequency drift > 0.0015  Wave Factor>.05  SD of Log R ratio>.35 and baf.drif>.0015  Log R ratio and B-allele frequency errors | 2  1  1  3 | 0  0  0  0 |
|  | Ethnicity | 0 | 1 |
|  | Relatedness | 0 | 3 |
|  | Total exclusions | 10 | 8 |
| **Final sample** |  | 585 | 328 |

^a^MBCFS: Mayo Breast Cancer Family Study; Mayo VTE: Venous thromboembolism Case-Control Study
