# Supplementary material for: The association of copy number variation and percent mammographic density
Source: BMC Res Notes. 2015 Jul 8;8:297. doi: 10.1186/s13104-015-1212-y (PMC4494822; doi:10.1186/s13104-015-1212-y)
Supplement: Additional file 3: — 48 candidate regions were identified in the data reduction step of the analysis (described in Supplementary Figure 1). Subsequently, five regions were identified as having a statistically-significant association with percent density in MBCFS. [file 13104_2015_1212_MOESM3_ESM.docx]

**Supplementary Table 2.** 48 candidate regions were identified in the data reduction step of the analysis (described in Supplementary Figure 1). Subsequently, five regions were identified as having a statistically-significant association with percent density in MBCFS^a^

| Chromosome | Start Position | End Position | # Probes passing the data reduction step (Supplementary Figure 1) | # significant probes from permutation testing |
| --- | --- | --- | --- | --- |
| 1 | 246817063 | 246861733 | 16 | 0 |
| 2 | 56507917 | 56509045 | 17 | 0 |
| 2 | 106059574 | 106062279 | 16 | 0 |
| 2 | 146579364 | 146593351 | 17 | 0 |
| 2 | 180122079 | 180124180 | 19 | 0 |
| 2 | 180124789 | 180131428 | 21 | 0 |
| 3 | 53003023 | 53013826 | 16 | 0 |
| **3** | **163995377** | **164008284** | **17** | **17** |
| **3** | **164030569** | **164108060** | **13** | **13** |
| 4 | 186678853 | 186681017 | 17 | 0 |
| 4 | 187330534 | 187337378 | 20 | 0 |
| 5 | 151495149 | 151498778 | 16 | 0 |
| 6 | 19150071 | 19157236 | 15 | 0 |
| 6 | 32627136 | 32630367 | 13 | 0 |
| 6 | 32630424 | 32658356 | 45 | 0 |
| 6 | 32658408 | 32668305 | 8 | 0 |
| 6 | 67098386 | 67099813 | 12 | 0 |
|  |  |  |  |  |
| 6 | 103852259 | 103894820 | 18 | 0 |
| 6 | 114330763 | 114331668 | 14 | 0 |
| 6 | 126225113 | 126228337 | 17 | 0 |
| 6 | 152431765 | 152434254 | 18 | 0 |
| 6 | 165644729 | 165651913 | 18 | 0 |
| 7 | 93158476 | 93177711 | 23 | 0 |
| 8 | 120225195 | 120229949 | 16 | 0 |
| **8** | **133134063** | **133144009** | **6** | **6** |
| 8 | 144705480 | 144707338 | 17 | 0 |
| 10 | 95535526 | 95538433 | 11 | 0 |
| **11** | **11779614** | **11780713** | **17** | **17** |
| 11 | 11832323 | 11834906 | 10 | 0 |
| 11 | 55127597 | 55209499 | 37 | 0 |
| 12 | 15910153 | 15927659 | 18 | 0 |
| 12 | 33606133 | 33608065 | 18 | 0 |
| 12 | 56298500 | 56366092 | 22 | 0 |
| 12 | 68573476 | 68576901 | 15 | 1 |
| 12 | 70472924 | 70476715 | 18 | 0 |
| 12 | 89012380 | 89015833 | 17 | 0 |
| 13 | 98052451 | 98055147 | 18 | 0 |
| 13 | 107745089 | 107749787 | 18 | 0 |
| 16 | 1363034 | 1364059 | 14 | 0 |
| 17 | 11352423 | 11353031 | 11 | 0 |
| 17 | 45173820 | 45247229 | 18 | 0 |
| **17** | **47649105** | **47667700** | **8** | **3** |
| 18 | 56823235 | 56823680 | 6 | 0 |
| 19 | 61558394 | 61560857 | 17 | 0 |
| 20 | 1509224 | 1542273 | 14 | 0 |
| 21 | 23272470 | 23432532 | 58 | 0 |
| 22 | 32110897 | 32112594 | 17 | 0 |
| Total |  |  | 829 | 57 |

^a^MBCFS: Mayo Breast Cancer Family Study

The number of probes within each initial region are obtained via the “Data Reduction and Analysis” step (Supplementary Figure 1). The significant by permutation testing are obtained via “Permutation Testing and Define Significance” (supplementary Figure 1).
