# Supplementary material for: The association of copy number variation and percent mammographic density
Source: BMC Res Notes. 2015 Jul 8;8:297. doi: 10.1186/s13104-015-1212-y (PMC4494822; doi:10.1186/s13104-015-1212-y)
Supplement: Additional file 9: — Coefficients and permutation based p-values for chromosome 3 and 17 using the discovery (MBCFS) and replication (Mayo VTE) cohorts. [file 13104_2015_1212_MOESM9_ESM.docx]

**Supplementary Table 3.** Coefficients and permutation based p-values for chromosome 3 and 17 using the discovery (MBCFS^a^) and replication (Mayo VTE^a^) cohorts

|  |  |  | MBCFS | | Mayo VTE | |
| --- | --- | --- | --- | --- | --- | --- |
| Chr |  | Position | Beta | Permutation p-value | Beta | Permutation p-value |
| 3 | rs12487989 | 163995377 | -0.11 | 0.000100 | 0.09 | 0.519248 |
| 3 | rs12488906 | 163996449 | -0.09 | 0.000100 | 0.10 | 0.520548 |
| 3 | rs9681771 | 163997393 | -0.11 | 0.000100 | 0.08 | 0.428357 |
| 3 | rs13062014 | 163997931 | -0.12 | 0.000100 | 0.09 | 0.454155 |
| 3 | rs11916098 | 163999374 | -0.09 | 0.000100 | 0.10 | 0.518148 |
| 3 | rs12185939 | 164000333 | -0.10 | 0.000100 | 0.08 | 0.522248 |
| 3 | rs28703661 | 164000498 | -0.10 | 0.000100 | 0.08 | 0.468653 |
| 3 | rs11923230 | 164001556 | -0.09 | 0.000100 | 0.11 | 0.517148 |
| 3 | rs11926853 | 164001668 | -0.09 | 0.000100 | 0.08 | 0.515448 |
| 3 | rs13086592 | 164002485 | -0.12 | 0.000100 | 0.08 | 0.453755 |
| 3 | rs12490361 | 164003254 | -0.10 | 0.000100 | 0.09 | 0.517548 |
| 3 | cnvi0111568 | 164004033 | -0.11 | 0.000100 | 0.11 | 0.487651 |
| 3 | rs9881842 | 164004443 | -0.13 | 0.000100 | 0.11 | 0.429557 |
| 3 | rs34394037 | 164006079 | -0.10 | 0.000100 | 0.13 | 0.469753 |
| 3 | rs1990726 | **164006187** | **-0.14** | **0.000100** | **0.13** | **0.000100** |
| 3 | rs13096689 | 164007284 | -0.12 | 0.000100 | 0.09 | 0.452255 |
| 3 | cnvi0068652 | 164008284 | -0.08 | 0.000100 | 0.10 | 0.487051 |
| 3 | rs13079279 | 164030569 | -0.12 | 0.000100 | 0.10 | 0.458254 |
| 3 | rs2041929 | 164037785 | -0.10 | 0.000100 | 0.10 | 0.471153 |
| 3 | rs2080523 | 164043283 | -0.09 | 0.000100 | 0.08 | 0.470153 |
| 3 | rs13327576 | 164051690 | -0.11 | 0.000100 | 0.09 | 0.447855 |
| 3 | cnvi0116513 | 164054566 | -0.11 | 0.000100 | 0.08 | 0.482952 |
| 3 | rs13090788 | 164059338 | -0.10 | 0.000100 | 0.07 | 0.454455 |
| 3 | rs12696170 | 164065358 | -0.10 | 0.000100 | 0.13 | 0.454555 |
| 3 | cnvi0111556 | 164078452 | -0.09 | 0.000100 | 0.06 | 0.484952 |
| 3 | rs9856048 | 164084306 | -0.08 | 0.000100 | 0.05 | 0.426557 |
| 3 | rs13066863 | 164090410 | -0.09 | 0.000100 | 0.07 | 0.449355 |
| 3 | rs9755769 | 164096351 | -0.10 | 0.000100 | 0.10 | 0.431357 |
| 3 | rs9831290 | 164102076 | -0.11 | 0.000100 | 0.15 | 0.429757 |
| 3 | cnvi0050222 | 164108060 | -0.10 | 0.000100 | 0.09 | 0.488151 |
|  |  |  |  |  |  |  |
| 17 | rs1531993 | **47649105** | **-1.49** | **0.000100** | **0.41** | **0.006599** |
| 17 | rs11867974 | **47649397** | **-0.37** | **0.000100** | **-0.67** | **0.006599** |
| 17 | rs12601661 | **47651778** | **0.72** | **0.000100** | **0.07** | **0.006599** |
| 17 | rs10514997 | 47654020 | 1.34 | 0.000200 | -0.26 | 0.527447 |
| 17 | rs10514999 | 47655029 | 1.84 | 0.000200 | -1.46 | 0.444356 |
| 17 | rs16951144 | 47658336 | 1.14 | 0.000300 | 0.83 | 0.440756 |
| 17 | rs10515000 | 47659558 | 0.35 | 0.000200 | 0.49 | 0.447355 |
| 17 | rs12936458 | 47667700 | 1.75 | 0.000200 | 1.83 | 0.456354 |
